# Methicillin-Resistant *Staphylococcus aureus* Associated with a Dental Abscess in a Captive Jaguar (*Panthera onca*): A Case Report

**DOI:** 10.3390/vetsci13070724

**Published:** 2026-07-22

**Authors:** Florin Dan Simiz, Doru Morar, Cristina Văduva, Simina Velescu, Liviu Todescu, Bogdan Alexandru Florea, Daniela Elena Brăslașu, Corina Marina Kracunovic, Janos Degi, Diana Maria Degi

**Affiliations:** 1 Faculty of Veterinary Medicine, University of Life Sciences “King Mihai I” from Timișoara, Calea Aradului No. 119, 300645 Timișoara, Romania; florinsimiz@usvt.ro (F.D.S.); dorumorar@usvt.ro (D.M.); cristina.vaduva@usvt.ro (C.V.); simina.velescu@usvt.ro (S.V.); bogdan-alexandru.florea.fmv@usvt.ro (B.A.F.); corina.kracunovic@usvt.ro (C.M.K.); degi.diana-maria.fbira@usvt.ro (D.M.D.); 2 Sibiu Zoological Garden, Calea Dumbrăvii No. 142, 550399 Sibiu, Romania; todesculiviu@gmail.com; 3 Faculty of Veterinary Medicine, University of Agronomic Sciences and Veterinary Medicine of Bucharest, Splaiul Independenței nr.105, Sector 5, 050097 Bucharest, Romania; danabraslasu@gmail.com

**Keywords:** MRSA, *Staphylococcus aureus*, jaguar, *Panthera onca*, dental abscess, antimicrobial resistance, One Health, zoo wildlife, captive felids, MALDI-TOF MS

## Abstract

MRSA is an important antimicrobial-resistant pathogen reported in a wide range of animal species; however, information regarding its occurrence in captive large felids remains limited. This report describes the detection and characterization of MRSA associated with a dental abscess in a captive jaguar (*Panthera onca*) from a zoological collection in Romania. A 10-year-old male jaguar presented with clinical signs suggestive of a localized oral infection. Samples collected from the dental lesion, nasal mucosa, external ear canal, and skin surface were subjected to bacteriological examination. Bacterial isolates were identified using conventional microbiological methods and Matrix-Assisted Laser Desorption/Ionization Time-of-Flight Mass Spectrometry (MALDI-TOF MS). Antimicrobial susceptibility testing was performed using the VITEK^®^ 2 system, while molecular confirmation was achieved by PCR targeting the *nuc*, *mecA*, and *mecC* genes. All isolates were identified as *S. aureus* and demonstrated a methicillin-resistant phenotype. PCR confirmed the presence of the species-specific *nuc* gene and the methicillin-resistance determinant *mecA*, while *mecC* was not detected. The isolates exhibited resistance to multiple antimicrobial classes, including β-lactams, fluoroquinolones, and macrolides, while remaining susceptible to linezolid, vancomycin, teicoplanin, rifampicin, and tigecycline. This case documents the occurrence of MRSA in a captive jaguar and highlights the value of integrated microbiological and molecular investigations for the diagnosis and characterization of resistant bacterial infections in zoological species.

## Simple Summary

Methicillin-resistant *Staphylococcus aureus* (MRSA) is an important pathogen in both human and veterinary medicine. Reports describing MRSA in captive wild felids remain uncommon. This case report describes the isolation of MRSA from a dental abscess and additional anatomical sites in a captive jaguar (*Panthera onca*) from a Romanian zoological collection. Bacterial identification was confirmed by conventional microbiological methods, MALDI-TOF MS, and PCR detection of the *nuc* and *mecA* genes. The isolates exhibited a multidrug-resistant phenotype consistent with MRSA. This report contributes additional information regarding MRSA occurrence in captive wildlife and highlights the importance of microbiological investigation of infectious lesions in zoological species.

## 1. Introduction

MRSA is an important opportunistic pathogen affecting both humans and animals and is recognized as a major antimicrobial resistance concern worldwide [1,2,3,4]. Although MRSA has been extensively investigated in companion animals, livestock, and veterinary hospital settings, reports involving captive wildlife remain relatively uncommon [3,5].

Although MRSA has been extensively investigated in humans, livestock, companion animals, and veterinary healthcare settings, comparatively little information is available regarding its occurrence in captive wildlife. Zoological collections represent unique environments where close interactions between animals, veterinary personnel, caretakers, and contaminated surroundings may facilitate the circulation of antimicrobial-resistant bacteria. Consequently, captive wild animals have increasingly been recognized as potential participants in the epidemiology of MRSA within the One Health framework [3,5,6,7,8].

Large felids maintained in zoological collections may be exposed to bacterial pathogens through close management, veterinary procedures, environmental contamination, or contact with caretakers [5,9]. However, information regarding MRSA infections in captive jaguars (*Panthera onca*) remains scarce, and available data on antimicrobial-resistant staphylococci in captive wild felids are still limited [3,5].

Reports describing MRSA infections in captive large felids remain exceptionally limited. Most available studies involving zoo carnivores have focused on bacterial surveillance or the detection of zoonotic pathogens rather than detailed microbiological characterization of clinical MRSA infections. Although bacterial infections have been reported in captive lions and other large felids, microbiologically confirmed MRSA infections in jaguars (*Panthera onca*) have rarely been documented. Consequently, information regarding their clinical presentation, antimicrobial susceptibility profiles, and molecular characteristics remains scarce [3,5].

Against this background, the present report describes the isolation and characterization of methicillin-resistant *S. aureus* recovered from a dental abscess and additional anatomical sites in a captive jaguar. By combining conventional bacteriological methods, MALDI-TOF MS identification, antimicrobial susceptibility testing, and PCR detection of the *nuc*, *mecA*, and *mecC* genes, this report contributes additional evidence regarding MRSA occurrence in captive wildlife and expands the limited body of literature available for captive large felids [3,5,8].

## 2. Materials and Methods

### 2.1. Clinical Presentation of the Jaguar

The patient was an adult intact male jaguar (*Panthera onca*), approximately 10 years of age and weighing 87 kg, originating from the Sibiu Zoological Garden, Romania (GPS coordinates: 45.7623° N, 24.1231° E). The animal was in good nutritional status and adequate body condition (Body Condition Score 3/5), with normal muscle mass and no evidence of emaciation or obesity. It presented clinical signs suggestive of a localized bacterial infection, including lethargy, reduced activity, decreased appetite, and signs of discomfort during feeding. General physical examination revealed no clinically significant abnormalities other than those associated with the oral lesion. Physical examination was performed under general anesthesia. Cardiovascular and respiratory parameters remained stable throughout the procedure and were continuously monitored, including heart rate, respiratory rate, rectal temperature, peripheral oxygen saturation (SpO_2_), capillary refill time, and mucous membrane color. No clinically significant abnormalities were detected outside the oral cavity.

**Figure 1:**
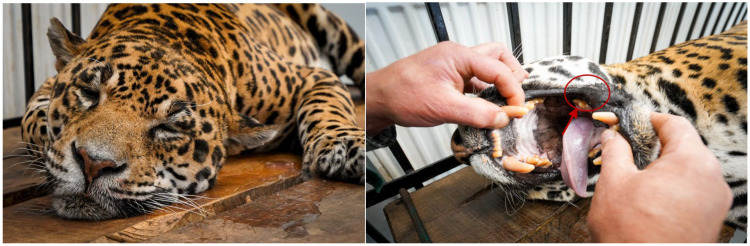
Close-up view of the oral cavity of the sedated jaguar showing the dental abscess (indicated by the arrow), characterized by localized gingival inflammation and purulent exudate adjacent to the affected tooth, consistent with an odontogenic infection.

Detailed oral examination revealed marked gingival inflammation surrounding the affected tooth, accompanied by an open purulent dental abscess, localized soft tissue swelling, and moderate gingival recession. Gentle manipulation of the lesion resulted in the release of purulent exudate and elicited signs of oral pain. Mild unilateral facial swelling and hypersalivation were also observed, while the remaining oral mucosa appeared macroscopically unremarkable, with no additional oral lesions detected. These clinical findings were consistent with an odontogenic infection and strongly supported the diagnosis of a dental abscess (Figure 1).

According to the medical records provided by the zoological collection, the animal had not received systemic antimicrobial treatment before sample collection.

**Figure 2:**
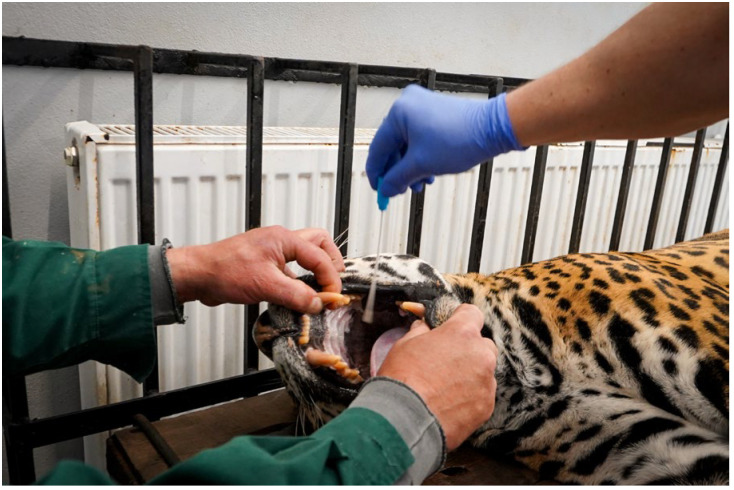
Collection of a microbiological sample from the oral cavity of the sedated jaguar using a sterile swab under controlled clinical conditions.

**Figure 3:**
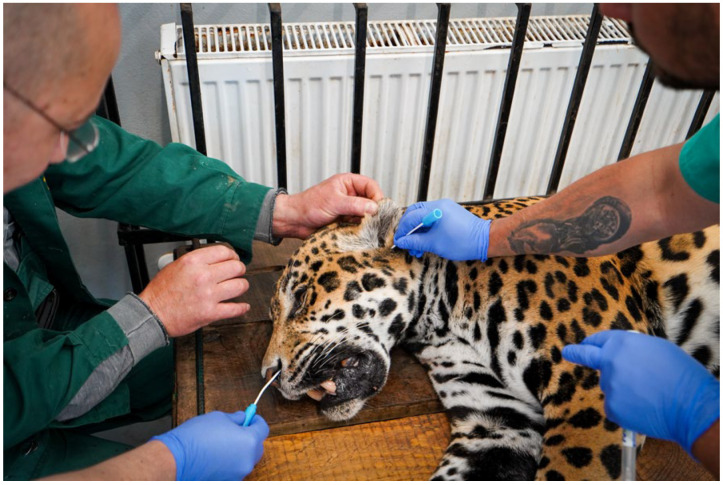
Collection of a microbiological sample from the nasal region of the sedated jaguar using a sterile swab under controlled clinical conditions.

**Figure 4:**
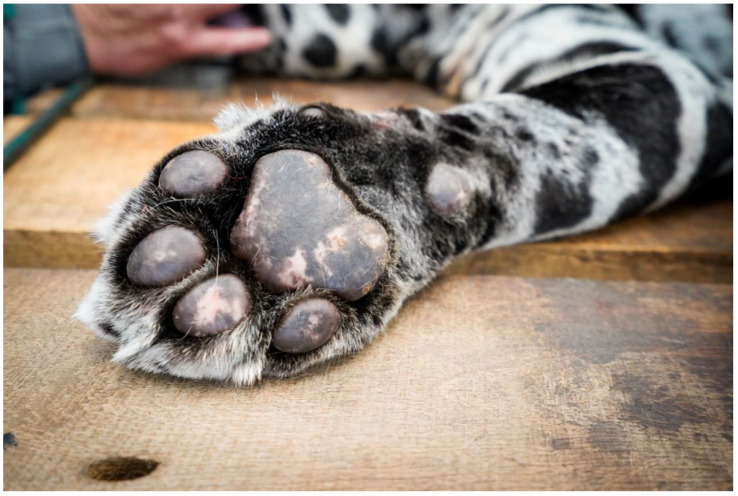
Collection of a cutaneous microbiological sample from the sedated jaguar using a sterile swab for bacteriological examination.

Microbiological samples were collected from the dental lesion, which was considered the primary site of infection, as well as from the nasal mucosa, external ear canal, and skin surface using sterile swabs. To evaluate the possible distribution of bacterial colonization, additional samples were collected from the nasal mucosa, external ear canal, and skin surface. Before initiation of any therapeutic intervention, microbiological specimens were aseptically collected from the dental lesion and the additional anatomical sites using sterile swabs, as illustrated in Figure 2, Figure 3 and Figure 4. The procedure was performed under controlled conditions with appropriate animal restraint and the use of personal protective equipment to ensure operator safety and sample integrity. All specimens were subsequently transported under appropriate conditions for bacteriological culture, antimicrobial susceptibility testing, and molecular analysis.

Therefore, the suspected dental abscess was evaluated both as a localized infectious process and as a possible source or consequence of pathogen transmission within the zoological environment. This integrated perspective supported subsequent microbiological investigation and epidemiological assessment of potential transmission pathways.

All clinical procedures, diagnostic investigations, and sample collection activities included in the present study were performed following signed informed owner consent and institutional collaboration agreements concluded with the Faculty of Veterinary Medicine, Timisoara (328/11.09.2024), in accordance with the provisions of Romanian Law No. 160/1998 regarding the organization and practice of the veterinary profession, as well as the Decision of the National Council of the Romanian College of Veterinarians No. 34/01.12.2012 concerning informed consent for veterinary medical procedures.

### 2.2. Anesthetic Protocol

For safe clinical examinations, dental extraction, and microbiological sample collection, the jaguar was immobilized using a standardized anesthetic protocol for large felids. Premedication consisted of medetomidine (0.05 mg/kg) and ketamine (3 mg/kg), administered remotely by darting. Following induction, anesthesia depth and physiological parameters, including heart rate, respiratory rate, rectal temperature, peripheral oxygen saturation (SpO_2_), and reflex responses, were continuously monitored. Oxygen supplementation and thermoregulatory support were provided throughout the procedure. After the clinical examination, sample collection, and dental extraction, anesthesia was reversed by intramuscular administration of atipamezole at five times the medetomidine dose. Recovery was uneventful, with the animal regaining normal posture and responsiveness without complications. The anesthetic protocol ensured safe restraint, minimized stress and cardiopulmonary complications, and facilitated the collection of high-quality diagnostic specimens while protecting both the animal and veterinary personnel.

Following microbiological sampling, the affected tooth was extracted under general anesthesia using standard veterinary dental extraction techniques after elevation of the gingival tissues and luxation of the periodontal ligament. The alveolus was thoroughly debrided and irrigated with sterile saline solution before routine closure of the surgical site. Postoperative management included analgesic and anti-inflammatory therapy together with systemic trimethoprim–sulfamethoxazole, selected according to the antimicrobial susceptibility results. The animal recovered uneventfully from anesthesia, the surgical site healed without complications, and progressive improvement in appetite and feeding behavior was observed during the follow-up period.

### 2.3. Bacterial Isolation, Species Identification, and Antimicrobial Susceptibility Testing

Clinical swab samples collected from the jaguar (dental abscess, nasal mucosa, external ear canal, and skin surface) were processed under standard bacteriological conditions for the isolation and presumptive identification of staphylococci, following conventional diagnostic microbiology procedures [10]. The samples were inoculated onto 5% sheep blood agar and mannitol salt agar and incubated aerobically at 37 °C for 24–48 h. Colonies showing morphology compatible with *Staphylococcus* spp. were selected for further investigation.

One representative isolate from each positive anatomical site was selected for further characterization, resulting in a total of four *S. aureus* isolates: one from the dental abscess, one from the nasal mucosa, one from the external ear canal, and one from the skin surface.

Following incubation, presumptive *S. aureus* colonies were identified based on their characteristic morphology, appearing as smooth, circular, convex, opaque, cream to golden-yellow colonies. Representative colonies displaying these characteristics were selected and subjected to conventional biochemical testing, including catalase and coagulase assays, followed by Matrix-Assisted Laser Desorption/Ionization Time-of-Flight Mass Spectrometry instrument (MALDI-TOF MS; Bruker Daltonics GmbH & Co. KG, Bremen, Germany) identification.

Presumptive isolates were initially characterized using conventional microbiological methods. Gram staining was performed using the BD Gram Stain Reagents Kit (Becton Dickinson, Sparks, MD, USA). Catalase testing was performed using 3% hydrogen peroxide solution (Sigma-Aldrich, St. Louis, MO, USA), and coagulase testing was conducted using the Staphaurex™ Latex Agglutination Test (Thermo Fisher Scientific, Oxoid, Basingstoke, UK) and/or the BBL™ Rabbit Plasma Coagulase Test (Becton Dickinson, Sparks, MD, USA), according to the manufacturers’ instructions.

Final species-level identification was performed using Matrix-Assisted Laser Desorption/Ionization Time-of-Flight Mass Spectrometry (MALDI-TOF MS; Bruker Daltonics, Bremen, Germany), according to the manufacturer’s instructions. Isolates reliably identified as *S. aureus* were included in subsequent antimicrobial susceptibility testing and molecular analyses.

Antimicrobial susceptibility testing was performed using the VITEK^®^ 2 Compact system with AST-P658 cards (bioMérieux, Marcy-l’Étoile, France). The antimicrobial panel included benzylpenicillin, oxacillin, cefoxitin screen, gentamicin, ciprofloxacin, levofloxacin, erythromycin, clindamycin, tetracycline, trimethoprim/sulfamethoxazole, rifampicin, vancomycin, teicoplanin, linezolid, tigecycline, fusidic acid, and mupirocin, consistent with the results reported in Table 1. Phenotypic screening for methicillin resistance was additionally performed using cefoxitin 30 μg disks (Oxoid, Thermo Fisher Scientific, Basingstoke, UK), according to EUCAST disk diffusion methodology and the EUCAST Clinical Breakpoint Tables, version 15.0 (2025) [10].

*S. aureus* ATCC 25923 and *S. aureus* ATCC 43300 were used as reference quality-control strains for phenotypic susceptibility testing and molecular confirmation, according to the manufacturers’ instructions and standard laboratory procedures.

### 2.4. Molecular Confirmation of S. aureus and Methicillin Resistance Determinants

Presumptive *Staphylococcus* isolates recovered from the clinical specimens were subjected to molecular confirmation using PCR. The assay targeted the species-specific nuc gene and the methicillin-resistance-associated genes *mecA* and *mecC*.

Genomic DNA was extracted from fresh pure cultures grown overnight on 5% sheep blood agar using the DNeasy Blood & Tissue Kit (Qiagen, Hilden, Germany), according to the manufacturer’s instructions. DNA purity and concentration were assessed using a NanoDrop™ 2000 spectrophotometer (Thermo Fisher Scientific, Waltham, MA, USA).

Species-level confirmation of *S. aureus* was performed by amplification of the thermostable nuclease gene (*nuc*) using the primers described by Brakstad et al. [11]: forward 5′-GCGATTGATGGTGATACGGTT-3′ and reverse 5′-AGCCAAGCCTTGACGAACTAAAGC-3′, generating a 279 bp amplicon.

Methicillin resistance determinants were investigated by amplification of the *mecA* and *mecC* genes using previously described primers. The *mecA* gene was amplified using the primers described by Murakami et al. [12]: forward 5′-AAAATCGATGGTAAAGGTTGGC-3′ and reverse 5′-AGTTCTGCAGTACCGGATTTGC-3′, generating a 533 bp product. The *mecC* gene was amplified using the primers described by Stegger et al. [13]: forward 5′-GAAAAAAAGGCTTAGAACGCCTC-3′ and reverse 5′-GAAGATCTTTTCCGTTTTCAGC-3′, generating a 138 bp product.

PCR reactions were performed in a final volume of 25 μL containing DreamTaq Green PCR Master Mix (2×) (Thermo Fisher Scientific, Waltham, MA, USA), 0.4 μM of each primer, approximately 50 ng of template DNA, and nuclease-free water. Amplification was carried out in a Veriti™ 96-Well Thermal Cycler (Applied Biosystems, Foster City, CA, USA). The cycling protocol consisted of initial denaturation at 95 °C for 5 min, followed by 35 cycles of denaturation at 95 °C for 30 s, annealing at 55–58 °C for 30 s, and extension at 72 °C for 45 s, with a final extension at 72 °C for 5 min.

Amplification products were separated by electrophoresis on 1.5% agarose gels stained with GelRed^®^ (Biotium, Fremont, CA, USA). Bands were visualized under ultraviolet illumination using a Gel Doc™ EZ Imaging System (Bio-Rad Laboratories, Inc., Hercules, CA, USA) and interpreted according to the expected amplicon sizes with reference to a 100 bp DNA ladder (Thermo Fisher Scientific, Waltham, MA, USA).

Each PCR run included appropriate controls: *S. aureus* ATCC 43300 as the *mecA*-positive MRSA control, *S. aureus* ATCC 25923 as the methicillin-susceptible negative control, a *mecC*-positive control strain, and a no-template control to exclude contamination.

The MRSA phenotype was defined by resistance to oxacillin together with a positive cefoxitin screening test, according to the EUCAST Clinical Breakpoint Tables, version 15.0 (2025).

Isolates were considered confirmed MRSA when they were positive for *nuc* together with *mecA* and/or *mecC*. Molecular results were interpreted in conjunction with phenotypic susceptibility testing, particularly the cefoxitin disk diffusion test, following EUCAST interpretive criteria.

## 3. Results

### 3.1. Clinical Examination Results

Clinical examination revealed that the jaguar was in a moderately reduced general condition, presenting with lethargy and decreased responsiveness. Oral inspection identified a pronounced dental lesion characterized by an open abscess, associated with local inflammation, soft tissue swelling, and signs of pain upon manipulation. Hypersalivation was also noted, supporting the presence of oral discomfort. No severe respiratory or neurological abnormalities were observed during the examination. The animal’s body condition appeared adequate, with no evident signs of systemic deterioration; however, the localized infection suggested a potential source for bacterial dissemination and warranted further microbiological investigation.

### 3.2. Bacteriological Identification Results

Colonies compatible with *Staphylococcus* spp. were recovered from all sampled sites, including the dental abscess, nasal mucosa, external ear canal, and skin surface. Presumptive isolates from each sample were Gram-positive cocci, catalase-positive, and coagulase-positive, consistent with *S. aureus*. A total of four representative isolates, one from each sampled anatomical site, were selected for further characterization and analyzed by MALDI-TOF MS. All four isolates were identified as *S. aureus* by MALDI-TOF MS, with identification scores above 2.0 for each isolate, supporting reliable high-confidence species-level identification.

### 3.3. Molecular Confirmation Results

PCR analysis was performed on representative *S. aureus* isolates recovered from the dental abscess, nasal mucosa, external ear canal, and skin surface. All tested isolates were positive for the species-specific *nuc* gene, yielding the expected 279 bp amplicon, thereby confirming their identification as *S. aureus*. Molecular screening for methicillin resistance demonstrated amplification of the *mecA* gene, with the expected 533 bp product, in the tested isolates. No amplification of the *mecC* gene was detected.

These molecular findings were consistent with the phenotypic cefoxitin resistance profile and supported the characterization of the isolates as MRSA. However, as no molecular typing method was performed, these results do not allow conclusions regarding the clonality of the isolates.

Representative agarose gel electrophoresis images supporting the PCR results for the *nuc*, *mecA*, and *mecC* genes are provided in Supplementary Figure S1.

### 3.4. Antimicrobial Susceptibility Testing Results

Antimicrobial susceptibility testing was performed on MRSA isolates recovered from the dental abscess, nasal mucosa, external ear canal, and skin surface. All isolates showed resistance to oxacillin and a positive cefoxitin screening result, consistent with a methicillin-resistant phenotype. Resistance to benzylpenicillin, ciprofloxacin, levofloxacin, and erythromycin was also observed in all isolates.

Differences between isolates were observed for gentamicin, clindamycin, tetracycline, fusidic acid, and mupirocin. All isolates remained susceptible to trimethoprim/sulfamethoxazole, rifampicin, teicoplanin, linezolid, and tigecycline. Vancomycin susceptibility was retained in all isolates, with MIC values ranging from 1 to 2 µg/mL. The complete MIC values and interpretations are presented in Table 1.

Legend: MIC, minimum inhibitory concentration; MRSA, methicillin-resistant *Staphylococcus aureus*; S, susceptible; R, resistant. Each column represents one MRSA isolate according to its anatomical origin.

Overall, all four isolates exhibited resistance to β-lactam antibiotics, fluoroquinolones, and erythromycin. Differences in antimicrobial susceptibility among isolates were observed for gentamicin, clindamycin, tetracycline, fusidic acid, and mupirocin. All isolates remained susceptible to trimethoprim/sulfamethoxazole, rifampicin, vancomycin, teicoplanin, linezolid, and tigecycline.

## 4. Discussion

This case report describes the isolation and characterization of MRSA associated with dental abscess and additional anatomical sites in a captive jaguar. Although MRSA was recovered directly from the purulent dental lesion, its isolation does not establish the organism as the primary causative agent of the abscess. *Staphylococcus aureus* may colonize mucosal and cutaneous surfaces in both humans and animals, and odontogenic infections may involve polymicrobial bacterial communities [3,8]. In the absence of anaerobic bacterial culture, histopathological examination, and broader microbiological investigation, the specific etiological contribution of MRSA to the development of the lesion cannot be conclusively determined. Nevertheless, its recovery from the purulent lesion in association with localized inflammation and oral pain supports the clinical relevance of this finding, while a direct causal relationship should not be inferred.

Published information on MRSA in captive large felids remains notably limited. Most investigations involving zoological felids have focused on general bacterial surveillance or the detection of zoonotic pathogens rather than on the detailed characterization of clinically apparent MRSA infections. For example, bacteriological investigations in captive African lions and other large felids have demonstrated exposure to and colonization by potentially pathogenic bacteria, highlighting the complex microbial environment of zoological collections [5]. In the broader wildlife context, *S. aureus*, including methicillin-resistant strains, has been detected in several animal species, supporting the concept that wildlife may participate in the epidemiology of antimicrobial-resistant staphylococci [1,3]. However, direct comparison with the present case is difficult because detailed clinical descriptions, antimicrobial susceptibility profiles, and molecular confirmation of MRSA in captive jaguars are rarely reported. The present case therefore adds clinical and microbiological information to a field in which species-specific data remain scarce.

The management of dental abscesses in captive large felids presents clinical challenges because detailed oral examination and therapeutic procedures generally require chemical immobilization. As with odontogenic infections in domestic species, effective management relies primarily on source control, including removal of the affected tooth, debridement of infected or inflammatory tissue, and appropriate local management of the alveolar site. In the present case, dental extraction and local debridement were performed after microbiological sampling. The isolation of MRSA highlights the additional value of bacterial culture and antimicrobial susceptibility testing in such cases, particularly when systemic antimicrobial therapy is considered. Susceptibility-guided treatment may help avoid empirical use of ineffective antimicrobials and supports antimicrobial stewardship in zoological medicine.

Phenotypic identification by conventional microbiological methods and MALDI-TOF MS was confirmed by PCR detection of the species-specific *nuc* gene and the methicillin-resistance determinant *mecA*, whereas *mecC* was not detected. These findings are consistent with previous reports identifying *mecA* as the predominant determinant of methicillin resistance in veterinary MRSA isolates [8,12,13]. MRSA was recovered from the dental lesion, nasal mucosa, external ear canal, and skin, demonstrating its presence at multiple anatomical sites. However, detection of the same resistance determinant and broadly comparable antimicrobial susceptibility profiles cannot establish clonal relatedness. Because *spa* typing, multilocus sequence typing (MLST), and whole-genome sequencing were not performed, it remains unknown whether these isolates belonged to the same MRSA clone or represented independent colonization events involving genetically distinct strains [14,15,16,17]. Therefore, the epidemiological relationship among the isolates cannot be determined from the present data.

The antimicrobial susceptibility profile observed in the present case is consistent with the multidrug-resistant phenotype commonly reported among MRSA isolates of veterinary origin. Resistance to β-lactam antimicrobials was expected in the context of *mecA*-mediated methicillin resistance; however, the concurrent resistance to fluoroquinolones and erythromycin demonstrates that the resistance phenotype extended beyond the β-lactam class. Similar multidrug-resistant profiles have been described in methicillin-resistant staphylococci isolated from domestic animals and veterinary clinical environments, although antimicrobial susceptibility patterns may vary according to the host species, previous antimicrobial exposure, and epidemiological setting [8,14,16]. Data regarding MRSA in wildlife remains comparatively limited; nevertheless, available reports indicate that both free-ranging and captive animals may harbor antimicrobial-resistant *S. aureus* strains exhibiting resistance to multiple antimicrobial classes [1,3]. In the present case, the differences observed among isolates in susceptibility to gentamicin, clindamycin, tetracycline, fusidic acid, and mupirocin further demonstrate phenotypic variability among MRSA recovered from different anatomical sites.

In contrast to the multidrug-resistant phenotype observed for several antimicrobial classes, all isolates remained susceptible to vancomycin, teicoplanin, and linezolid. The absence of phenotypic resistance to glycopeptides and linezolid is particularly relevant from an epidemiological and antimicrobial resistance surveillance perspective because these agents are critically important for the treatment of severe MRSA infections in human medicine. However, their preserved in vitro activity should not be interpreted as supporting their routine therapeutic use in zoological species. Rather, these findings emphasize the importance of antimicrobial susceptibility testing, susceptibility-guided treatment, and prudent antimicrobial selection, particularly when other active agents are available [4,8,16].

From a clinical perspective, the recovery of MRSA from the dental lesion and multiple additional anatomical sites highlights the need to consider both localized infection and concurrent bacterial colonization in captive large felids. The clinical role of MRSA in odontogenic infections in these species remains poorly defined; however, in the present case, its isolation directly from the purulent dental lesion, together with localized inflammation, soft tissue swelling, oral pain, hypersalivation, and impaired feeding, supports the clinical relevance of the MRSA isolation in this case. Nevertheless, these findings do not establish MRSA as the sole or primary etiological agent, particularly because anaerobic culture was not performed and odontogenic infections may involve polymicrobial bacterial communities.

Following extraction of the affected tooth, local debridement, and susceptibility-guided antimicrobial treatment, the jaguar showed progressive improvement in appetite and feeding behavior, with no immediate postoperative complications. This favorable clinical evolution supports the importance of effective source control combined with targeted antimicrobial therapy. In this context, bacterial identification and antimicrobial susceptibility testing may provide valuable information for therapeutic decision-making and antimicrobial stewardship in zoological medicine. However, the absence of long-term follow-up prevents conclusions regarding persistent MRSA colonization or recurrence.

From a One Health perspective, the detection of MRSA in a captive jaguar raises questions regarding the potential circulation of antimicrobial-resistant staphylococci at the animal–human–environment interface. In zoological settings, direct transmission may potentially occur during close contact associated with routine husbandry, animal restraint, clinical examination, or veterinary procedures. Indirect transmission may also involve contaminated hands, reusable equipment, fomites, or surfaces within animal enclosures and clinical areas [3,5,9,16]. Conversely, colonized animals may represent a potential source of environmental contamination or occupational exposure for caretakers and veterinary personnel. The recovery of MRSA from the nasal mucosa, external ear canal, and skin surface of the jaguar, in addition to the dental lesion, further highlights the potential for bacterial shedding from multiple anatomical sites. However, no microbiological screening of zoo personnel, other animals, or the enclosure environment was performed; therefore, neither the source nor the direction of potential MRSA transmission can be determined. These findings support an integrated surveillance approach combining clinical microbiology, targeted screening when epidemiologically indicated, environmental hygiene, appropriate use of personal protective equipment, and strict hand hygiene to limit the potential circulation of antimicrobial-resistant bacteria in zoological collections.

Several limitations should be acknowledged. This report describes a single clinical case, and comprehensive laboratory investigations, diagnostic imaging, and long-term clinical follow-up were not available. Moreover, microbiological investigation was primarily focused on *S. aureus*, and a comprehensive characterization of other aerobic and anaerobic bacterial species potentially involved in the dental lesion was not performed. Histopathological examination of the lesion and molecular typing of the isolates were also unavailable. These limitations precluded a comprehensive assessment of the potential polymicrobial nature of the infection and the broader reservoir of antimicrobial resistance determinants. Consequently, neither the primary etiological role of MRSA in the development of dental abscess nor the genetic relatedness of the isolates could be conclusively established.

Despite these limitations, the present report expands the limited literature on MRSA infections in large captive felids by providing combined clinical, microbiological, antimicrobial susceptibility, and molecular findings from a captive jaguar. The findings underscore the importance of integrating microbiological diagnostics and antimicrobial susceptibility testing into the clinical management of infectious diseases in zoological species and provide additional evidence regarding the occurrence and clinical relevance of MRSA in captive wildlife [3,5,17].

## 5. Conclusions

This case highlights the clinical relevance of considering MRSA and other antimicrobial-resistant bacteria in the diagnostic investigation of infectious lesions in captive wildlife. The recovery of MRSA from a dental abscess and multiple anatomical sites in a captive jaguar emphasizes the value of combining clinical examination with bacterial identification, antimicrobial susceptibility testing, and molecular confirmation. Susceptibility-guided antimicrobial selection, together with effective source control of odontogenic infections, may support appropriate therapeutic decision-making and antimicrobial stewardship in zoological medicine.

Beyond the individual clinical case, the detection of MRSA in a captive large felid underscores the importance of microbiological surveillance and appropriate infection control measures in zoological collections, where interactions among animals, caretakers, veterinary personnel, and the environment may influence the circulation of antimicrobial-resistant bacteria. However, the absence of molecular typing prevents determination of whether the MRSA isolates recovered from the different anatomical sites belonged to the same clone or represented independent colonization events. Further investigations incorporating *spa* typing, MLST, whole-genome sequencing, and broader epidemiological surveillance are needed to clarify the genetic relatedness, transmission dynamics, and clinical significance of MRSA in captive wildlife.

## Figures and Tables

**Table 1:** MIC values (µg/mL) and antimicrobial susceptibility profiles of four MRSA isolates recovered from different anatomical sites of a captive jaguar, determined using the VITEK^®^ 2 AST-P658 system.

Antimicrobial Agent	Dental Abscess Isolate	Nasal Mucosa Isolate	External Ear Canal Isolate	Skin Surface Isolate
Oxacillin	≥4 (R)	≥4 (R)	≥4 (R)	≥4 (R)
Cefoxitin screen	Positive (R)	Positive (R)	Positive (R)	Positive (R)
Benzylpenicillin	≥0.5 (R)	≥0.5 (R)	≥0.5 (R)	≥0.5 (R)
Gentamicin	≥16 (R)	≥16 (R)	1 (S)	1 (S)
Ciprofloxacin	≥4 (R)	≥4 (R)	≥4 (R)	≥4 (R)
Levofloxacin	≥4 (R)	≥4 (R)	≥4 (R)	≥4 (R)
Erythromycin	≥8 (R)	≥8 (R)	≥8 (R)	≥8 (R)
Clindamycin	≥4 (R)	≥4 (R)	0.5 (S)	0.25 (S)
Tetracycline	≥8 (R)	≥8 (R)	1 (S)	0.5 (S)
Trimethoprim/sulfamethoxazole	≤10 (S)	≤10 (S)	≤10 (S)	≤10 (S)
Rifampicin	≤0.5 (S)	≤0.5 (S)	≤0.5 (S)	≤0.5 (S)
Vancomycin	2 (S)	1 (S)	1 (S)	1 (S)
Teicoplanin	≤2 (S)	≤2 (S)	≤2 (S)	≤2 (S)
Linezolid	≤2 (S)	≤2 (S)	≤2 (S)	≤2 (S)
Tigecycline	≤0.5 (S)	≤0.5 (S)	≤0.5 (S)	≤0.5 (S)
Fusidic acid	≥2 (R)	≥2 (R)	0.5 (S)	0.5 (S)
Mupirocin	≥512 (R)	≥512 (R)	≤1 (S)	≤1 (S)

## Data Availability

The data presented in this study are available on reasonable request from the corresponding author. The data is not publicly available due to ethical and institutional restrictions.

## Supplementary Materials

The following supporting information can be downloaded at: https://www.mdpi.com/article/10.3390/vetsci13070724/s1, Figure S1: Representative agarose gel electrophoresis images of PCR amplification targeting the *nuc*, *mecA*, and *mecC* genes in *Staphylococcus aureus* isolates recovered from the captive jaguar. The expected amplicon sizes were 279 bp for *nuc*, 533 bp for *mecA*, and 138 bp for *mecC*. M, 100 bp DNA molecular weight marker; lanes 1–4, isolates recovered from the dental abscess, nasal mucosa, external ear canal, and skin surface, respectively; PC, positive control; NC, negative control; NTC, no-template control. All tested isolates showed amplification of *nuc* and *mecA*, whereas no *mecC* amplification was detected.

## Abbreviations

The following abbreviations are used in this manuscript:

Abbreviation	Full term
MRSA	Methicillin-resistant *Staphylococcus aureus*
MSSA	Methicillin-susceptible *Staphylococcus aureus*
MALDI-TOF MS	Matrix-Assisted Laser Desorption/Ionization Time-of-Flight Mass Spectrometry
PCR	Polymerase Chain Reaction
DNA	Deoxyribonucleic acid
AST	Antimicrobial Susceptibility Testing
MIC	Minimum Inhibitory Concentration
EUCAST	European Committee on Antimicrobial Susceptibility Testing
ATCC	American Type Culture Collection
MLST	Multilocus Sequence Typing
PPE	Personal Protective Equipment
bp	Base pairs
