# Supplementary material for: Methicillin-Resistant Staphylococcus aureus Associated with a Dental Abscess in a Captive Jaguar (Panthera onca): A Case Report
Source: Vet Sci. 2026 Jul 22;13(7):724. doi: 10.3390/vetsci13070724 (PMC13431599; doi:10.3390/vetsci13070724)
Supplement: Supplementary file 1 [file vetsci-13-00724-s001.zip › vetsci-4415920-supplementary.pdf]

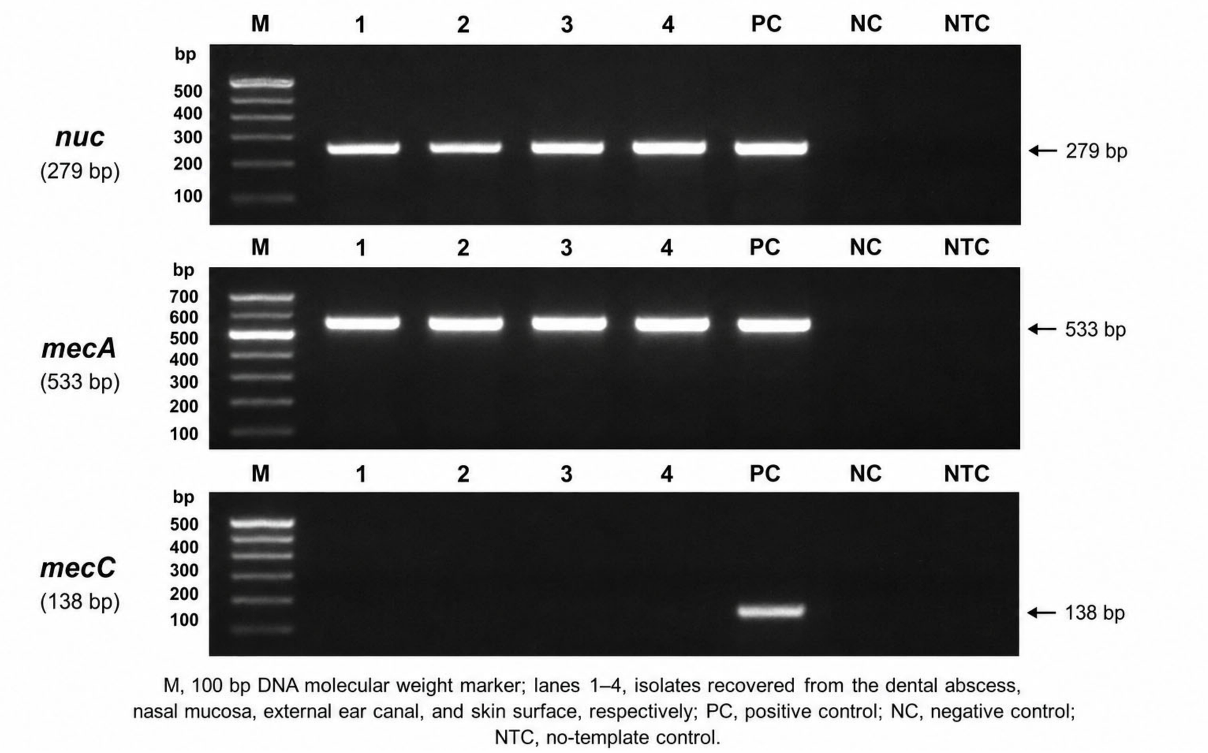

Figure S1. Representative agarose gel electrophoresis images of PCR amplification targeting the *nuc*, *mecA*, and *mecC* genes in *Staphylococcus aureus* isolates recovered from the captive jaguar. The expected amplicon sizes were 279 bp for *nuc*, 533 bp for *mecA*, and 138 bp for *mecC*. M, 100 bp DNA molecular weight marker; lanes 1–4, isolates recovered from the dental abscess, nasal mucosa, external ear canal, and skin surface, respectively; PC, positive control; NC, negative control; NTC, no-template control. All tested isolates showed amplification of *nuc* and *mecA*, whereas no *mecC* amplification was detected.
